# Red Light, Purple Light! Results of an Intervention to Promote School Readiness for Children From Low-Income Backgrounds

**DOI:** 10.3389/fpsyg.2019.02365

**Published:** 2019-10-22

**Authors:** Megan M. McClelland, Shauna L. Tominey, Sara A. Schmitt, Bridget E. Hatfield, David J. Purpura, Christopher R. Gonzales, Alexis N. Tracy

**Affiliations:** ^1^Human Development and Family Sciences, Oregon State University, Corvallis, OR, United States; ^2^Extension Family and Community Health Program, Oregon State University, Corvallis, OR, United States; ^3^Human Development and Family Studies, Purdue University, West Lafayette, IN, United States; ^4^Center for Mind and Brain, University of California, Davis, Davis, CA, United States

**Keywords:** self-regulation, executive function, intervention, school readiness, academic achievement

## Abstract

Considerable research has examined interventions that facilitate school readiness skills in young children. One intervention, *Red Light, Purple Light Circle Time Games* (RLPL*;* Tominey and McClelland, 2011; Schmitt et al., 2015), includes music and movement games that aim to foster self-regulation skills. The present study (*N* = 157) focused on children from families with low-income and compared the RLPL intervention (SR) to a revised version of RLPL that included literacy and math content (SR+) and a Business-As-Usual (BAU) control group. In both versions of the intervention, teachers were trained to administer the self-regulation intervention in preschool classrooms with coaching support. Although not statistically significant, children receiving either version of the intervention gained more in self-regulation on the Head-Toes-Knees-Shoulders (HTKS) over the preschool year compared to the BAU group (β = 0.09, *p* = 0.082, Cohen’s *d* = 0.31). Effect sizes were similar to previous studies (Schmitt et al., 2015; Duncan et al., 2018) and translated to a 21% difference in self-regulation over and above the BAU group at post-test. Furthermore, children participating in either version of the intervention gained significantly more in math across the school year compared to children in the BAU group (β = 0.14; *p* = 0.003, Cohen’s *d* = 0.38), which translated to a 24% difference in math over and above the BAU group at post-test. Results were somewhat stronger for the SR+ version, although effect sizes across intervention conditions were comparable. There were no statistically significant differences across groups for literacy skills. Results extend previous research and suggest that the RLPL intervention, which includes an explicit focus on self-regulation through music and movement games, may improve children’s self-regulation and math scores over the preschool year.

## Introduction

A disproportionate number of children with low self-regulation and academic skills at kindergarten entry are from families experiencing socioeconomic disadvantage (Evans and Rosenbaum, 2008; Wanless et al., 2011; Blair and Raver, 2015). Given existing school readiness gaps, it is critical to design programs that promote the development of self-regulation skills for children from diverse backgrounds. In recent years, numerous interventions have emerged that include self-regulation as part of more comprehensive programs, many that also include academic skills (e.g., PATHS, Tools of the Mind; Diamond et al., 2007; Domitrovich et al., 2007). Although many of these interventions have shown significant effects in improving aspects of children’s self-regulation, few have demonstrated substantive effects on self-regulation and early academic skills (Bierman et al., 2008; Raver et al., 2011), and others demonstrate no effects on self-regulation or academic outcomes (Farran et al., 2013; Morris et al., 2014). Moreover, comprehensive curricula require extensive training to implement with fidelity, which may help explain null, small, and moderate effect sizes. Although aligned with best practices for early childhood, the comprehensive approach to intervention can make it challenging, if not impossible, to determine what part of each program is most effective. In order to accommodate early childhood education programs that are likely to have limited resources and time to commit to professional development, it is critical to develop interventions where the impact of specific components can be tested in order to identify core elements that could be integrated with little time and at low-cost into existing comprehensive early childhood curricula.

The present study evaluated and compared the effectiveness of two versions of a teacher-implemented school readiness intervention called *Red Light, Purple Light Circle Time Games* (RLPL; McClelland and Tominey, 2015). One version of the program was a self-regulation only version (SR), and the other was a self-regulation plus math and reading version (SR+), which was informed by best practices to support reading and math development. Both were designed for teachers to administer in preschool classroom settings. Given the targeted nature of the intervention and that RLPL requires few resources to implement (e.g., materials found in typical early childhood classrooms, half day of professional development), the intervention can feasibly be implemented in classrooms to benefit self-regulation and early academic achievement.

### The Development of School Readiness

#### Self-Regulation

Self-regulation has been conceptualized across disciplines in many ways; however, it is commonly recognized as a multidimensional concept that incorporates emotion, cognition, and behavior (McClelland et al., 2010). The present study focuses on the aspects of self-regulation most relevant in classroom contexts, which are related to three underlying executive function (EF) cognitive processes: working memory, attentional or cognitive flexibility, and inhibitory control (Cameron Ponitz et al., 2009). Working memory refers to the ability to maintain and manipulate information (Gathercole, 2008); attentional or cognitive flexibility is the ability to sustain focus and adapt to changing goals (Rothbart and Posner, 2005); and inhibitory control includes stopping a dominant response in favor of a more appropriate one (Blair, 2003). Although each aspect of EF contributes to academic outcomes, evidence suggests that the integration of working memory, attentional or cognitive flexibility, and inhibitory control in children’s overt behavior is important for their success in early classroom contexts (McClelland and Cameron, 2012; Blair and Raver, 2015). In this study, we refer to *self-regulation* to capture children’s EF processes in real-world settings. Self-regulation emerges in early childhood and during this period, acquisition of these skills involves various environmental and developmental processes (Blair and Raver, 2015; McClelland et al., 2015). In addition, self-regulation has been shown to be a malleable set of skills that mediate the relation between early risk and academic success (Sektnan et al., 2010). Thus, targeting self-regulation prior to formal schooling may be one way to improve children’s school readiness.

#### Early Math Skills

Early math consists of skills and concepts that build upon one another and include domains such as numeracy (National Mathematics Advisory Panel, 2008; National Research Council [NRC], 2009). Early numeracy is comprised of skills related to counting and cardinality, quantity comparison, numeral knowledge, and more advanced mathematical (or arithmetic) operations (National Mathematics Advisory Panel, 2008; Purpura and Lonigan, 2013). These aspects of numeracy are critical for later mathematics skills according to many international benchmarks (Common Core State Standards Initiative, 2002; National Council of Teachers of Mathematics, 2006; Australia Curriculum Assessment Reporting Authority, 2013; Curriculum Development Council, 2017). Moreover, deficits in early mathematics skills are likely to lead to long-term difficulties (Aunola et al., 2004; Every Child a Chance Trust and KPMG, 2008; National Research Council [NRC], 2009).

#### Emergent Literacy Skills

Three components of emergent literacy measured in preschool are believed to form the foundation for the acquisition of literacy skills: oral language, phonological awareness, and print knowledge. Oral language is comprised of skills such as word knowledge, vocabulary, and understanding grammatical rules and word order (Storch and Whitehurst, 2002). Phonological awareness refers to children’s ability to detect and manipulate language through blending, matching, or removing parts of words (Wagner and Torgesen, 1987). Print knowledge includes children’s awareness of basic print conventions (i.e., letter names and sounds; Whitehurst and Lonigan, 1998). Children who enter school with difficulties in emergent literacy skills are likely to experience reading difficulties that persist over time (Storch and Whitehurst, 2002). Moreover, children with low levels of early reading skills are at elevated risk for needing special education services (Lentz, 1988).

#### Connections Between Self-Regulation and Academic Skills

A large body of research indicates that self-regulation is an important part of academic success in childhood, adolescence, and into adulthood (McClelland et al., 2006, 2007, 2013; Duckworth et al., 2010; Blair and Raver, 2015). In addition to empirical evidence indicating a strong predictive relation between self-regulation and academic achievement, interventions that aim to improve self-regulation have also shown significant effects on children’s math and literacy (Tominey and McClelland, 2011; Blair and Raver, 2014; Schmitt et al., 2015; Pandey et al., 2018) suggesting that self-regulation may be an important precursor for early achievement. In addition, children’s self-regulation has been found to be especially predictive of early math skills where children have to focus and pay attention, remember and execute step-by-step instructions, and demonstrate self-control, all of which are important for learning math (McClelland et al., 2014; Blair et al., 2015; Purpura et al., 2017). In addition, research suggests that relations between self-regulation and mathematics and literacy may be bidirectional and more complex than previously thought (Fuhs et al., 2014; Schmitt et al., 2017). The bidirectional connections between self-regulation, math, and literacy suggest the promise of an intervention that targets the integration of these skills.

#### The Role of Socio-Demographic Risk for Self-Regulation, Math, and Literacy

A large body of research documents negative relations between children’s socioeconomic risk and children’s academic outcomes (e.g., Duncan and Magnuson, 2005). Children from low-income households typically experience more difficulty with the development of math and literacy skills than children from middle-income families (Jordan et al., 1992). Recent work also documents the negative effects on children’s self-regulation (e.g., Wanless et al., 2011; Raver et al., 2012). In the United States, ethnic minorities, and particularly Spanish-speaking English language learners (ELLs) are more likely to experience elevated risks, such as poverty and low parent education levels (U. S. Census Bureau, 2011), which may negatively impact children’s outcomes. However, research suggests that self-regulation may be an important protective factor for children growing up from disadvantaged backgrounds (Obradovic, 2010; Sektnan et al., 2010). This suggests that promoting self-regulation for children at socio-demographic risk is important for successful learning outcomes in school.

### Existing School Readiness Interventions

A number of classroom-based interventions that specifically target self-regulation and early academic skills have demonstrated effectiveness. Examples of interventions include the preschool Promoting Alternative Thinking Strategies (PATHS) curriculum, which focuses on children’s problem solving skills, emotional awareness, social-emotional skills and self-control (Greenberg and Kusche, 1993; Kam et al., 2003; Domitrovich et al., 2007). There are also interventions that focus explicitly on improving preschoolers’ early math that have been shown to be effective such as Pre-K Mathematics (Starkey et al., 2004; Thomas et al., 2018) and Building Blocks (Clements and Sarama, 2007; Clements and Sarama, 2011). Finally, interventions designed to promote preschoolers’ emergent literacy have shown positive effects (Justice and Pullen, 2003; Farver et al., 2009; Justice et al., 2009).

Although these interventions have shown to be successful at improving children’s outcomes, they typically require in-depth training, time (for planning/professional development as well as for implementation), materials, and significant expense. Furthermore, many interventions target a range of skills, making it difficult to determine the specific mechanisms that are responsible for observed changes in self-regulation and academic achievement. For example, Head Start REDI (Research-Based, Developmentally Informed), emphasizes literacy, language and social-emotional skills and has been shown to be effective at improving children’s self-regulation and academic outcomes (Bierman et al., 2014; Sasser et al., 2017). Another program, Tools of the Mind, also focuses on early literacy and self-regulation with mixed results of its effectiveness (Farran et al., 2013; Blair and Raver, 2014). It is difficult, however, to identify which aspects of these interventions are most effective and none of the interventions reviewed target self-regulation *and* early math and emergent literacy.

### Red Light, Purple Light (RLPL) Intervention

The Red Light, Purple Light Intervention (RLPL) is a classroom-based, self-regulation intervention consisting of circle time, music and movement games that have been designed to systematically increase in cognitive complexity over 16 sessions (delivered twice a week for 8 weeks). The games are delivered in a large-group format in 15–20 min sessions (Tominey and McClelland, 2011; McClelland and Tominey, 2015; Schmitt et al., 2015). The games focus on the three aspects of EF (i.e., working memory, attentional or cognitive flexibility, and inhibitory control) and enable children to practice self-regulation in a classroom setting (i.e., children play the games in a large group, such as during circle time).

The intervention consists of five games (one played per session), which are repeated multiple times over the course of the intervention, but with increasing levels of complexity in the variation of the game that is reintroduced. An example of one of the intervention games is *Red Light, Purple Light*, which is a variation of the childhood game Red Light, Green Light. In this game, the teacher acts as a stoplight and holds up different colors of construction paper circles that represent stop and go. The first time the game is introduced, the teacher asks children to respond to green (“go”) and red (“stop”) circles, with children performing different actions when the teacher holds up green (e.g., stomp, clap, hop) and stopping or freezing when the teacher holds up red. The game increases in complexity where the teacher adds colors (e.g., orange and purple) and children are asked to respond to opposite cues. Children are also given the opportunity to lead, choosing colors and actions for their classmates to respond to.

In the SR+ version of the games, literacy (print knowledge and phonological awareness) and math (counting and cardinality and numerical knowledge) content is embedded into the cues children are asked to respond to. For example, when playing *Red Light, Purple Light*, instead of responding to colors, children are shown a circle with a number written on it. In addition to responding to the color (e.g., clapping when they see blue, stomping when they see orange), children are shown a number card and asked to perform the action as many times as represented on the card (e.g., if teacher holds up a number 4, children clap 4 times, counting from 1–4 together as they clap). When playing the *Sleeping Game*, children pretend to go to sleep when the teacher sings the “Sleeping Song” and then wake up and act out the animal named by the teacher. In the SR+ version of the game, the teacher emphasizes print knowledge and phonological awareness (e.g., “When you wake up, pretend to be the first animal that I say that starts with an ‘m.’ Snake! Does that start with an ‘m?’ Mouse!”). In another, teachers show a picture of the animal with the printed word underneath (teachers use pictures in the SR version, but without words). A detailed manual with information about the games and sessions is also given to teachers (see section Materials and Methods).

Like each of the RLPL games, Red Light, Purple Light targets children’s EF skills where children have to listen and remember instructions (i.e., working memory), successfully move from one rule to another (i.e., attentional flexibility), and do the opposite as part of a game (i.e., inhibitory control). As the intervention progresses, new games are introduced and games are repeated with additional rules introduced to increase cognitive complexity. In each game, children respond to visual and/or oral cues and are often asked to respond to opposite cues. In the SR+ version of the intervention, the cues children are asked to respond to before choosing their actions relate specifically to literacy or math.

The self-regulation-only (SR) version of the RLPL intervention has been evaluated in two randomized controlled trials (RCTs) administered by researchers in preschool classrooms and one RCT where the games were delivered by teachers (Tominey and McClelland, 2011; Schmitt et al., 2015; Duncan et al., 2018). In one study, participation in the intervention was associated with improvement in self-regulation for children with low initial scores on self-regulation [e.g., a score of zero on the Head-Toes-Knees-Shoulders (HTKS) measure], and gains in literacy for the overall sample in comparison with a control group (Tominey and McClelland, 2011). Results from a larger study with children from disadvantaged backgrounds (i.e., enrolled in Head Start) found that participation in the intervention was significantly related to gains in self-regulation for the overall sample and gains in math for English language learners (Schmitt et al., 2015). In each of these studies, researchers with previous classroom experience led the games in early childhood classroom settings. A recent study examined the RLPL games delivered by teachers and included as part of a summer school readiness program (Duncan et al., 2018). In the RCT part of the study, children who participated in the summer program with RLPL games experienced significant improvement in self-regulation compared to children who participated in the summer program without exposure to RLPL games. There were no significant effects of intervention participation on math or literacy at the end of the program. However, when children were followed into the fall of kindergarten, participation in the summer program with the RLPL intervention was related to greater change in self-regulation, math, and literacy scores from the beginning of the intervention to the fall of kindergarten compared with children’s expected development using a separate longitudinal sample.

An important aspect of the RLPL intervention is the focus on ease-of-use and feasibility: the games require little training to implement, few materials (those readily available in early childhood classroom settings), and have been reported to be engaging for children with a range of developmental levels and needs (Tominey and McClelland, 2013). Moreover, the games were developed to be implemented as part of daily activities (i.e., large group time) and embedded in existing classroom curricula.

### Theory of Change

Preschool is an ideal time to implement a self-regulation intervention because of the rapid development in the prefrontal cortex, an area associated with self-regulation and EF skills (Blair, 2002). For most children, the preschool classroom is the first early learning environment in which they are asked to demonstrate self-regulation skills. Moreover, preschool is an important time for developing the early math and emergent literacy skills that are related to academic achievement in later elementary and high school (Duncan et al., 2007; Clements and Sarama, 2011).

Conceptually, our theory of change hypothesizes that promoting self-regulation would help children develop skills required to effectively take advantage of learning opportunities, including those that focus on math and literacy. With the added version of the intervention (SR+), the present study tested the idea that embedding academic content would not only help children develop the self-regulation skills needed to benefit from these learning opportunities, but also to extend that learning to those specific learning contexts. The self-regulation games require children to pay attention to, remember, and follow increasingly complex sets of rules through multiple exposure and repeated practice.

In addition to teaching and practicing self-regulation, the SR+ components of the classroom games provide additional complexity and were hypothesized to impact self-regulation more strongly than the SR components alone. For example, given the strong relations between self-regulation and early academic skills, it is possible that targeting these skills together would have the greatest benefit on self-regulation (Duncan et al., 2007). We focused on aspects of early math (counting, cardinality, and numeral knowledge) and emergent literacy (phonological awareness and print knowledge) that are most strongly related to early self-regulation (Purpura et al., 2017). Previous evidence has supported the effectiveness of the intervention on self-regulation and academic outcomes, especially math, in young children (e.g., Tominey and McClelland, 2011; Schmitt et al., 2015). Thus, we anticipated that the self-regulation games would result in significant positive impacts on self-regulation and academic outcomes, particularly math, compared to the BAU delayed intervention group.

### The Present Study

The present study evaluated an intervention that explicitly focuses on self-regulation (attentional flexibility, working memory, and inhibitory control) and compared the core curriculum with an enhanced version of the curriculum with embedded early math (counting, cardinality and numeral knowledge) and literacy (phonological awareness and print knowledge) components, given that these skills are foundational for academic success.

In summary, the specific aims of this study were to:

(1) Examine if there are significant effects of the self-regulation intervention (testing for effects of each version of the intervention: SR and SR+) on self-regulation over the preschool year in children from low-income backgrounds.
(2) Examine if there are significant effects of the self-regulation intervention (SR and SR+ versions) on children’s academic achievement (early literacy and math skills) over the preschool year.

We compared two versions of the intervention (SR and SR+) with a Business-As-Usual (BAU) delayed intervention group on children’s school readiness skills (self-regulation and academic achievement) over the preschool year. One version included the self-regulation games from our previous research (e.g., Tominey and McClelland, 2011; McClelland and Tominey, 2015; Schmitt et al., 2015; SR), and one version (SR+) included enhanced early math and literacy components added to the original self-regulation games. Given the strong relations between early self-regulation and academic achievement (and math in particular), it was possible that targeting these skills together would have the greatest benefit on self-regulation (Duncan et al., 2007). We anticipated that both versions of the intervention would result in significant positive impacts on self-regulation and academic skills, especially math, compared to the BAU delayed intervention group. Further, the SR+ version was expected to lead to stronger effects than the BAU condition or the SR-only intervention on early math and literacy skills because this version explicitly aimed to incorporate mathematical thinking and emergent literacy into the self-regulation games.

## Materials and Methods

### Participants

Children, parents, and teachers for the current study were from a study focused on developing, refining, and testing the promise of a self-regulation intervention. The initial sample consisted of 188 children (52% female) from low-income families who were participating in Head Start, a U.S. preschool program for low-income families. Children were recruited from 13 Head Start classrooms across nine sites in the Pacific Northwest of the United States. Children and families were recruited through consent forms distributed in enrollment packets during the summer prior to the start of preschool.

In the fall of the preschool year (time 1), a total of 188 children were eligible to participate. At time 2 in the spring of the preschool year, 157 children from the initial sample participated. This was an attrition rate of 17%. Children who did not participate in the post-test did not significantly differ from the other children who completed the study in terms of gender, maternal education, English language learner (ELL) status or on any of the measures described below at pre-test (*p* > 0.05), but did differ in terms of age. Children who did not participate in the post-test session were more likely to be younger than children who did participate, *t*(184) = 3.10, *p* = 0.002. All of the analyses described below were conducted using the data from the 157 children who contributed at least partial data at both pre-test and post-test.

Parents’ education level ranged from 2 to 17 years (*M* = 11.27, *SD* = 2.30). Children were eligible to participate in the study if they were between the ages of 3–5 and attending, or planning to attend, one of the 13 target classrooms. At pre-test, children had an average age of 51 months (range = 38–62 months, *N* = 41 3-year-olds, 99 4-year-olds, 17 5-year-olds), and at post-test had an average age of 58 months (range = 44 – 68 months, *N* = 12 3-year-olds, 80 4-year-olds, 65 5-year-olds).

More than half of the sample of children and families identified as Latino (58%), 26% identified as White, 7% Pacific Islander, 6% African American, and 2% reported other for ethnicity. Information from the consent form (child’s home language) identified 62 children (33%) as ELLs. Spanish-speaking research assistants administered the Pre Language Assessment System (preLAS) at pre-test and post-test to determine whether a child should receive direct assessments in English or Spanish (Duncan and De Avila, 1985–1987). If children did not pass the preLAS, and their home language was not Spanish, they were not administered any assessments at that time point (*n* = 2). Eight teachers (all female) across 13 classrooms and seven sites consented to participate. Five teachers had separate morning and afternoon classrooms (*n* = 10 classrooms); three teachers taught in either morning or afternoon (*n* = 3 classrooms).

### Procedure

In the fall (pre-test) and spring (post-test) of the preschool year, all direct assessments were administered using trained research assistants. Assessments were given in 10–15 min sessions inside the classroom in a quiet area or in a hallway. All assessments were completed in 2–3 classroom visits, depending on child absences, and the order of assessments was counterbalanced. Children identified as ELL’s were assessed by Spanish-speaking research assistants at pre and post-test, whether or not children passed the preLAS. Parents and teachers completed demographic questionnaires.

#### Pre-test

Direct assessments of self-regulation and early academic achievement were administered to children in the fall of the preschool year.

#### Intervention

To prevent contamination, block randomization occurred at the teacher level in the winter so that teachers leading more than one classroom (i.e., teachers with a morning and an afternoon class) delivered the same condition in each classroom. Eight teachers were included in the study who were supporting a total of 13 classrooms (half-day classrooms). Five teachers taught across a full day (one morning session; one afternoon session) and three teachers taught half-day only (one morning session or one afternoon session). Of the five full-day teachers, two were randomly assigned to the SR group (2 teachers; 2 classrooms each = 4 classrooms total), two were randomly assigned to the SR+ group (2 teachers; 2 classrooms each = 4 classrooms total), and one was randomly assigned to the control group (1 teacher; 2 classrooms). Of the three half-day teachers, each was randomly assigned to one of the conditions (SR, SR+, and control). In total, five classrooms were assigned to each intervention condition (SR or SR+) and three were randomly assigned to the control. Of the three sites with intervention classrooms, only one site had all classrooms receiving the same condition of the intervention (SR+).

The training of the intervention was consistent aside from the difference in content that included either self-regulation content (SR) or self-regulation with embedded literacy and math content (SR+). Learning goals were created for each of the sessions to demonstrate how session content related to the specific SR or SR+ intervention condition. The SR condition did not include any explicit instruction related to early math or literacy skills so learning goals only related to the three aspects of self-regulation (inhibitory control, attentional flexibility, and working memory). In the SR+ condition learning goals related to those same three components of self-regulation, but also included an explicit focus on emergent literacy skills (e.g., embedding dialogue related to early literacy into game play) and early math skills (e.g., counting together the number of intervention sessions 1 to 16; emphasizing the number of times actions were performed and using spoken numbers to cue children). During the intervention half-day training, teachers were asked not to share information across classrooms or with other teachers.

##### Teacher training

Intervention classroom teachers (*n* = 6) attended a half-day training led by two master trainers, one training for SR classrooms, and one for SR+ classrooms held in separate locations. Teachers participating in the SR training learned about the importance of self-regulation in the classroom along with the core elements of the self-regulation intervention and had an opportunity to use their training manual and materials. Teachers participating in the SR+ training received a similar training to the SR classroom, but also received information on embedding math and literacy content into the intervention games.

##### Intervention implementation

Through an iterative development process working with a set of master teachers, the research team refined the RLPL training materials and classroom kits prior to implementation, including detailed session plans and refinement of fidelity of implementation surveys (i.e., surveys teachers were asked to complete following each session related to implementation). Teachers participating in this RCT received a comprehensive intervention training manual and classroom kit at training. For some classrooms, both lead and assistant teachers were present, however, only lead teachers (unless absent) implemented intervention sessions in the classroom. Following the training, 100% of teachers reported on training evaluation surveys that they agreed or strongly agreed they felt prepared to play the games in their classrooms. Implementation began 1 week after the training, during winter of the preschool year. Teachers implemented the RLPL intervention in their classrooms, twice a week over 8 weeks for 15–20 min during large group circle time. Children in the control classrooms engaged in the daily routines and curricula activities that came before study participation (business-as-usual).

#### Dosage, Fidelity, and Feasibility of Implementation

To capture dosage of the intervention, teachers completed an attendance sheet after each session (2x a week). Fidelity of implementation was monitored each week through teacher reported daily logs completed at the end of each session. To assess, feasibility, teachers were asked to rate their own and their students’ enjoyment of the games played in each session, if the manual and materials were helpful, and overall length, difficulty, and prep time for each session. In addition, all intervention classroom teachers (*n* = 6) received coaching support and met six times with their coach throughout the intervention implementation. Teachers were coached on three dimensions of implementation fidelity- adherence, quality, and responsiveness. As part of the coaching process, teachers recorded intervention sessions to be reviewed during their one-on-one coaching session the following week. Additionally, over the course of the intervention, 43 videos were collected from intervention and BAU classrooms for the research team to use and code for fidelity. The video coding team attended a 3-h training on video coding processes (i.e., the importance of objectivity) and the coding rubric created by the coaching development team. Coders attended weekly meetings and provided codes on a series of master coded videos to obtain reliability. Once group reliability was achieved, all intervention videos were double coded and consensus codes were used to assess fidelity. These videos were also used to explore the presence of similar self-regulation games in BAU classrooms as well as to code for fidelity of implementation – adherence, quality, and responsiveness across all intervention classrooms.

#### Post-test

In the spring of the preschool year, the same direct assessments on self-regulation and academic achievement were administered to children. All research assistants were blind to children’s treatment and control group participation.

### Measures

#### Parent Demographic Questionnaire

Parents completed a survey in English or Spanish with questions about children’s age, gender, child care experiences, health, and parent and family characteristics such as years of education completed, work status, and household size.

#### Language Screener

The Simon Says and Art Show subtests of the preLAS were used to determine language of assessment. Simon Says is a measure of receptive language and Art Show is a measure of expressive language assessing naming and descriptive vocabulary. These two subtests of the preLAS have been demonstrated to have strong reliability and validity in Spanish-speaking preschool aged children (Rainelli et al., 2017). If children did not pass the preLAS, and parent identified as Spanish-speaking, they were assessed in Spanish.

### Direct Measures of Self-Regulation

The Head-Toes-Knees-Shoulders-Revised (HTKS-R) task was used to assess children’s self-regulation and taps aspects of attention, working memory, and inhibitory control (McClelland et al., 2014). The task has four sections and is a complex version of the HTKS (McClelland et al., 2014) for children ages 3–8. In the first section, children are asked to say the opposite of what is instructed. In the next section, children are told to touch their head (or toes) when asked to touch their toes (or head). Then, in the following section, both rules are included (head/toes opposite and knees/shoulders opposite). In the last section, children are still doing the opposite, but the rules are switched with different pairings. There were a total of 58 items across the 4 sections. Items are scored 0 for an incorrect response, 1 for a self-corrected response, and 2 for a correct response and overall scores range from 0 to 116. The HTKS-R and HTKS have demonstrated strong reliability and validity in diverse samples around the world including significant relations to other tasks measuring aspects of self-regulation and EF (e.g., Wanless et al., 2011; McClelland et al., 2014). The measure has also been sensitive to intervention effects, showing significant change in response to participation in self-regulation interventions when compared with children in a control group (Tominey and McClelland, 2011; Schmitt et al., 2015; Duncan et al., 2018; Landis et al., 2018; Upshur et al., 2019). In the current sample, the HTKS-R demonstrated adequate to strong internal reliability (Cronbach’s α = 0.96 at pre-test and 0.97 at post-test).

Children’s inhibitory control was assessed using the Day-Night Stroop task (Gerstadt et al., 1994). Children are presented with 16 cards with pictures of a sun or moon and asked to say the opposite (e.g., “day” for a moon and “night” for a sun). The measure has demonstrated strong reliability in research (Rhoades et al., 2009; McClelland et al., 2014). In the current sample, the Day-Night Stroop task demonstrated strong internal reliability (Cronbach’s α = 0.90 at pre-test and 0.91 at post-test).

### Academic Outcomes

#### Emergent Literacy Skills

The Letter-Word Identification subtest of the Woodcock Johnson Tests of Achievement (Woodcock et al., 2001) or The Batería III Woodcock- Muñoz (Muñoz-Sandoval et al., 2005) was used to assess emergent literacy. Research has shown high reliability and validity (α > 0.80) for all of the subtests (Woodcock and Mather, 2000; Schrank et al., 2005). In the present study, *W* scores were used in the analyses, which are standardized based on the average performance for a child at a particular age (Jaffe, 2009). *W* scores are appropriate for emergent literacy skills. The Letter-Word Identification subtest measures children’s letter skills and developing word-decoding skills with strong reliability and validity. Reliability for English-speaking preschool children ranges between 0.98–0.99 and 0.84-0.98 for Spanish-speaking children.

#### Early Math Skills

Children’s early math skills were assessed using the Preschool Early Numeracy Skills Screener (PENS; Purpura et al., 2015). This numeracy task consists of 24 items that are ordered by difficulty, progressing from the easiest items to the most difficult. The PENS assesses aspects of numeracy including set comparisons, numeral comparisons, one-to-one correspondences, number order, numeral identification, ordinality, and number combinations. Children receive 1 point for each correct answer. If a child responds incorrectly to three items in a row, the assessment ends. The assessment takes approximately 5 min to administer. In the current sample, the PENS demonstrated adequate internal reliability (Cronbach’s α = 0.91 at pre-test and 0.92 at post-test).

#### Control Variables

Children’s age in months, gender, parent education in years, ELL status, and baseline self-regulation or academic achievement scores were used as control variables in models. Previous research has shown these variables to be related to children’s self-regulation and early academic achievement (Cameron Ponitz et al., 2009; Wanless et al., 2011).

## Results

### Analytic Strategy

All analyses were conducted using Stata 15.1 (StataCorp, 2017). Due to the hierarchical structure of the data with children nested within different classrooms, we first evaluated whether a multilevel framework was necessary to accurately test the effects of the two versions of the intervention in comparison with a BAU control. The ICCs from the intercept-only models for both the self-regulation outcomes (ICC range:0.02 – 0.05) and the academic outcomes (ICC range: <0.001 – 0.001) were small, but were within a range where accounting for the nested structure of the data is appropriate (Hox et al., 2010). Thus, we utilized clustered robust standard errors for all analyses described below which adjust standard errors for the nested structure of the data.

We ran separate, but parallel analyses for each of the self-regulation and academic outcomes. All models included children’s performance at pre-test on the outcome variable, their age, gender, ELL status, and parent level of education when evaluating the effect of the different interventions. For each model, we also utilized an intent to treat (ITT) analysis (Fisher et al., 1990) where children’s scores were analyzed as part of their assigned intervention group regardless of whether or not they were present for all aspects of their assigned intervention group. To calculate the estimated effect sizes of the interventions on the outcome variables, the estimated mean differences over and above the control group from each of the final models were divided by overall standard deviation of the outcome variable at pre-test (Feingold, 2009).

#### Missing Data

For the 157 children in the analyses, data were missing for a small percentage of children on the HTKS-R at pre-test (8%) and post-test (10%), Day-night at pre-test (3%) and post-test (6%) WJ-Letter-word at pre-test (3%) and post-test (8%) and on PENS at pre-test (3%) and post-test (11%). Data on individual measures were typically missing due to child absences at one of the testing sessions or other extraneous factors. For all of the analyses described below, these data were assumed to be missing at random (MAR; Little and Rubin, 2002). Although there are no definitive tests of the MAR assumption (Baraldi and Enders, 2010), we assessed whether missingness on any of the variables was due to any auxiliary variables available in the dataset using logistic regression and no significant predictors emerged. Thus, we concluded an MAR assumption was valid (Acock, 2012).

To account for MAR data in our analyses, we ran path models with a full information maximum likelihood (FIML) estimator in Stata 15.1 (Muthén and Muthén, 2012; StataCorp, 2017) for all of the final models described below. A FIML estimator utilizes all available data in the analysis and provides more unbiased estimates compared to traditional missing data techniques such as pairwise or listwise deletion (Enders and Bandalos, 2001).

### Fidelity of Implementation

On average, participating children attended 14 sessions across both intervention groups and 95% of participating children attended at least 10 intervention sessions. As noted above, 43 videos were collected from intervention and BAU classrooms and coded for fidelity (adherence, quality, and responsiveness). All participating teachers delivered 100% of the intervention sessions, in order, and on the dates scheduled (two times per week for 8 weeks). Videos of intervention classrooms indicated that teachers implemented the intervention effectively (e.g., played the correct games, modeled appropriate behaviors) and adhered to the condition of the intervention they were trained in. Coders did not observe any deviations from the session guides and learning objectives included in the training manual. All participating classrooms (BAU and intervention classrooms) used Creative Curriculum. A review of the curricula and lesson plans used by BAU classrooms along with video observations also confirmed that teachers in BAU classrooms were not playing self-regulation games of a similar nature to those in either version of the intervention as part of their typical practice.

### Descriptive Statistics

Bivariate correlations between all these variables are also presented in Table 1. Descriptive statistics for all direct assessments at pre-test and post-test and all control variables are presented in Table 2. Two-sample *t*-tests (Table 1) were conducted to assess for any differences at pre-test between children assigned to the BAU control group and children assigned to either of the intervention conditions. Although random assignment was utilized, significant baseline differences were found on children’s performance on the HTKS-R and on the PENS at pre-test and marginally significant differences were found on children’s performance on Day-Night at pre-test. There were also significant differences in the proportion of ELL children in the BAU control group and the two intervention groups. Specifically, children in classrooms with teachers randomly assigned to the control group had higher baseline scores on each of these measures than children in classrooms with teachers randomly assigned to treatment groups. Thus, fall baseline scores on the HTKS-R, Day-Night, or PENS were included as control variables in models predicting these corresponding outcomes in the spring. In addition, ELL status was included as a control variable in all models.

**TABLE 1:** Pairwise correlations of variables.

**Variables**	**1**	**2**	**3**	**4**	**5**	**6**	**7**	**8**	**9**	**10**	**11**	**12**	**13**
(1) Age	−												
(2) Gender	0.18^∗^	−											
(3) Fall ELL Status	–0.11	–0.04	−										
(4) Parent Edu	–0.13	0.12	–0.37^∗∗^	−									
5. Fall HTKS	0.33^∗∗∗^	0.00	–0.37^∗∗∗^	0.15	−								
(6) Spring HTKS	0.46^∗∗∗^	0.04	–0.33^∗∗∗^	0.23	0.56^∗∗∗^	−							
(7) Fall Day-Night	0.08	–0.10	0.10	–0.15	0.17^∗^	0.10	−						
(8) Spring Day-Night	0.09	–0.07	–0.03	–0.05	0.24^∗∗^	0.22^∗^	0.30^∗∗∗^	−					
(9) Fall Letter-word	0.40^∗∗∗^	–0.04	0.12	–0.11	0.27^∗∗^	0.33^∗∗∗^	0.19^∗^	0.12	−				
(10) Spring Letter-word	0.30^∗∗∗^	–0.03	–0.07	–0.07	0.38^∗∗∗^	0.36^∗∗∗^	0.21^∗^	0.15	0.64^∗∗∗^	−			
(11) Fall PENS	0.54^∗∗∗^	0.03	–0.33^∗∗∗^	0.20	0.62^∗∗∗^	0.62^∗∗∗^	0.16^∗^	0.18^∗^	0.48^∗∗∗^	0.56^∗∗∗^	−		
(12) Spring PENS	0.51^∗∗∗^	0.10	–0.41^∗∗∗^	0.04	0.52^∗∗∗^	0.68^∗∗∗^	0.15	0.29^∗∗∗^	0.48^∗∗∗^	0.56^∗∗∗^	0.72^∗∗∗^	−	
(13) SR Treatment	0.12	0.02	0.17^∗^	–0.36^∗∗^	–0.06	–0.04	0.06	–0.03	0.12	0.14	–0.09	0.00	−
(14) SR + Treatment	–0.11	–0.11	0.06	0.12	–0.09	–0.01	0.06	0.08	−0.19^∗^	−0.19^∗^	–0.07	–0.01	–0.62^∗∗∗^

*^∗^*p* < 0.05, ^∗∗^*p* < 0.01, ^∗∗∗^*p* < 0.001.*

**TABLE 2:** Means (SD) of each variable by intervention condition.

	**Overall sample (*N* = 157)**				**Difference tests^b^**	
		
	**BAU (*N* = 37)**	**SR (*N* = 59)**	**SR+ (*N* = 61)**	**Any treatment (*N* = 120)**	***t***	***g***
Age	51.73 (6.90)	52.84 (5.49)	51.04 (6.45)	51.92 (6.04)	*t*(155) = 0.17	0.03
Gender	0.59	0.51	0.43	0.47	χ^2^(1) = 2.66	
Fall ELL-Status	0.05	0.36	0.30	0.33	χ^2^(1) = 10.76^∗∗∗^	
Parent Education	12.26 (1.43)	10.13 (3.23)	11.62 (1.31)	10.83 (2.61)	*t*(68) = 2.26^∗^	0.60
Fall HTKS	29.84 (23.80)	21.36 (23.92)	20.30 (17.13)	20.83 (20.72)	*t*(142) = 2.10^∗^	0.42
Spring HTKS	41.52 (27.61)	36.74 (31.22)	38.05 (32.54)	37.43 (31.79)	*t*(145) = 0.67	0.13
Fall Day-Night	15.63 (11.44)	18.72 (9.31)	18.73 (9.68)	18.73 (9.46)	*t*(151) = 1.62^†^	0.31
Spring Day-Night	21.6 (10.34)	22.09 (9.45)	23.38 (8.85)	22.75 (9.13)	*t*(146) = 0.63	0.12
Fall Letter-Word	314.94 (28.9)	315.5 (25.98)	305.65 (21.11)	310.49 (24.04)	*t*(151) = 0.92	0.18
Spring Letter-Word	331.09 (25.83)	333.29 (22.72)	323.33 (22.13)	328.08 (22.86)	*t*(142) = 0.65	0.13
Fall PENS	6.71 (5.26)	4.59 (4.32)	4.69 (4.31)	4.64 (4.29)	*t*(151) = 2.35^∗∗^	0.46
Spring PENS	8.94 (5.69)	8.75 (5.87)	8.69 (5.20)	8.72 (5.51)	*t*(139) = 0.20	0.04

*^*b*^Difference tests (Hedge’s g) calculated between the BAU group and both treatment groups combined (Any Treatment). ^†^*p* < 0.10, ^∗^*p* < 0.05, ^∗∗^*p* < 0.01, ^∗∗∗^*p* < 0.001.*

### Hypothesis Testing

Parallel path models utilizing all available data at post-test were conducted for each of the outcome variables which are presented below. Estimated effects of treatment condition and all other covariates in the final models are included in Table 3.

**TABLE 3:** Estimated effects for intervention conditions vs. BAU control on self-regulation, mathematics, and literacy at post-test (*N* = 157).

**Variable**	**Self-regulation**				**Math**				**Literacy**			
	**β**	***SE***	***P-value***	***Cohen’s d***	**β**	***SE***	***P-value***	***Cohen’s d***	**β**	***SE***	***P-value***	***Cohen’s d***
**BAU versus Any Treatment**												
Pre-test Score	0.40	0.06	< 0.001		0.59	0.07	< 0.001		0.64	0.07	< 0.001	
Age	0.35	0.05	< 0.001		0.15	0.05	0.004		0.06	0.10	0.534	
ELL- Status	–0.09	0.08	0.246		–0.22	0.04	< 0.001		–0.13	0.09	0.172	
Gender	–0.03	0.06	0.680		0.03	0.07	0.676		0.02	0.07	0.791	
Parent Education	0.22	0.06	< 0.001		–0.05	0.05	0.374		0.01	0.13	0.924	
Intervention ^*a*^	0.09	0.07	0.082	0.31	0.14	0.05	0.003	0.38	0.01	0.05	0.401	0.03
**BAU versus SR and SR+**												
Pre-test Score	0.39	0.07	< 0.001		0.60	0.06	< 0.001		0.62	0.07	< 0.001	
Age	0.34	0.05	< 0.001		0.15	0.05	0.002		0.06	0.10	0.524	
ELL-Status	–0.10	0.07	0.158		–0.22	0.04	< 0.001		–0.13	0.09	0.171	
Gender	–0.02	0.06	0.695		0.03	0.07	0.657		–0.03	0.07	0.722	
Parent Education	0.18	0.08	0.023		–0.07	0.05	0.215		0.03	0.13	0.816	
Intervention												
SR^a^	0.09	0.09	0.168	0.25	0.14	0.06	0.016	0.34	0.06	0.05	0.108	0.12
SR+^a^	0.11	0.07	0.066	0.32	0.17	0.06	0.004	0.38	–0.02	0.08	0.609	0.03

*All estimates are from a path model accounting for missing data (estimator = FIML) and the nested data structure (robust clustered standard errors). ^*a*^*P*-values for Intervention effects are one-tailed tests.*

#### Self-Regulation Outcomes

We first tested whether there were any effects of either condition of the intervention (SR and SR+ versions combined) on children’s self-regulation over the preschool year, given that both conditions included the same underlying self-regulation components. As shown at the top of Table 3, children receiving either version of the intervention demonstrated higher self-regulation on the HTKS-R at post-test compared to the business as usual group. Although results were not statistically significant, they indicated a significant trend (β = 0.09, *p* = 0.082). In addition, the estimated mean difference of children’s self-regulation at post-test over and above the BAU group (*M* = 6.81 points, Cohen’s *d* = 0.31, 95% CI: −0.10 – 0.72) were similar to gains made on the HTKS in previous intervention studies (Schmitt et al., 2015; Duncan et al., 2018) and consisted of a 21% difference over and above the BAU group at post-test.

**FIGURE 1:**
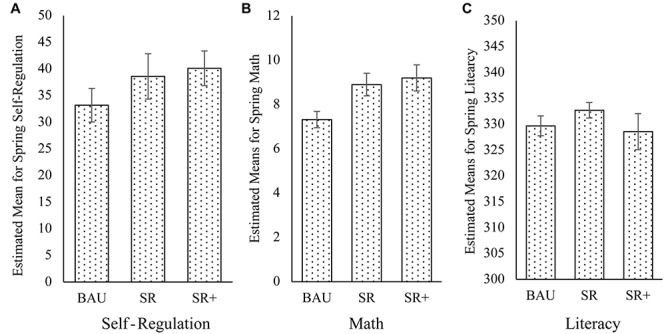
Estimated means at post-test in **(A)** Self-Regulation, **(B)** Math, **(C)** Literacy (±SE) the BAU, SR, and SR+ groups controlling for initial scores at pre-test, age, ELL-status, gender and parental education.

When evaluating the individual intervention types as shown at the bottom of Table 3, gains in self-regulation for the SR+ group over the BAU group were larger (*M* = 6.93 points, Cohen’s *d* = 0.32, 95% CI: −0.26 – 0.76) with a trend for a significant difference over and above the BAU group (β = 0.11, *p* = 0.066), whereas estimated gains on the HTKS-R in the SR group were smaller (*M* = 5.41 points, Cohen’s *d* = 0.25, 95% CI: −0.11 – 0.75), and were not significantly different from the BAU group (β = 0.09, *p* = 0.168). Although there was a trend for the effect of the SR+ version to be larger than the SR version, the difference between the two intervention groups was not statistically significant (*p* = 0.394, *d* = 0.07, 95% CI: −0.44 – 0.58). Children’s estimated mean performance on the HTKS-R at post-test is illustrated in Figure 1A. Differences in children’s performance on the Day-Night task at post-test were not significantly different between children in either of the intervention groups and the BAU control group.

#### Academic Achievement Outcomes

Second, we tested whether there were any significant effects of either version of the intervention (SR and SR+) on children’s academic achievement (early literacy and math skills) over the preschool year. As shown on Table 3, children receiving either intervention demonstrated significantly higher math scores on the PENS at post-test compared to children in the BAU group (β = 0.14; *p* = 0.003). The estimated mean difference in children’s math ability on the PENS (*M* = 1.75 points, Cohen’s *d* = 0.38, 95% CI:0.15 – 0.61) was equivalent to a 24% difference over and above the BAU group.

Children in the SR+ version of the intervention demonstrated significantly higher math scores on the PENS at post-test (β = 0.17, *p* = 0.003) compared to children in the BAU group (*M* = 1.76 points, Cohen’s *d* = 0.38, 95% CI:0.15 – 0.61) with a similar significant difference (β = 0.14, *p* = 0.016) for children in the SR version of the intervention (*M* = 1.57; Cohen’s *d* = 0.34, 95% CI:0.07 – 0.62). Estimated mean differences in children’s math ability on the PENS did not differ significantly from the SR intervention group or SR+ intervention group versions of the intervention, *p* = 0.698, Cohen’s *d* = 0.03, 95% CI: −0.26 – 0.40. Children’s estimated mean performance on the PENS at post-test is illustrated in Figure 1B.

Finally, we tested whether either version of the intervention demonstrated any significant effects on children’s early literacy skills. As shown on the top of Table 3, children receiving either version of the intervention did not demonstrate any significant difference in their early literacy skills compared to the BAU group at post-test (β = 0.01, *p* = 0.924). As shown in Figure 1C, when examining the intervention groups individually, neither the SR intervention (β = 0.06, *p* = 0.108) nor the SR+ intervention (β = −0.02, *p* = 0.609) demonstrated any significant difference compared to the BAU group.

#### Exploratory Analyses

We also conducted a series of exploratory analyses to assess whether there were any significant effects of the interventions (SR and SR+ versions) on children’s self-regulation (measured on the HTKS) over the preschool year for children who started out with initially low levels of self-regulation on the HTKS as in previous studies (e.g., Tominey and McClelland, 2011). First, we tested for an interaction between the effect of the intervention and whether children started with low initial levels of self-regulation, and then conducted a subgroup analysis examining the effect of the intervention for children with low self-regulation. However, given the available sample size in the current study, results reported below should be interpreted with caution.

In a previous study (e.g., Tominey and McClelland, 2011), children were determined to have low levels of self-regulation if they initially received a zero on the HTKS. The HTKS-R contains all the same components of the HTKS but adds a downward extension to capture more variability in children with low self-regulation. To capture the limited variability of children with low-levels of self-regulation on the HTKS-R and align with previous studies, we coded children as having low self-regulation if they did not get at least 4 out of the 6 possible points on the initial practice questions for Part 1 of the measure. At pre-test, 74% of children (*n* = 116) did not meet the initial threshold on the HTKS-R.

**FIGURE 2:**
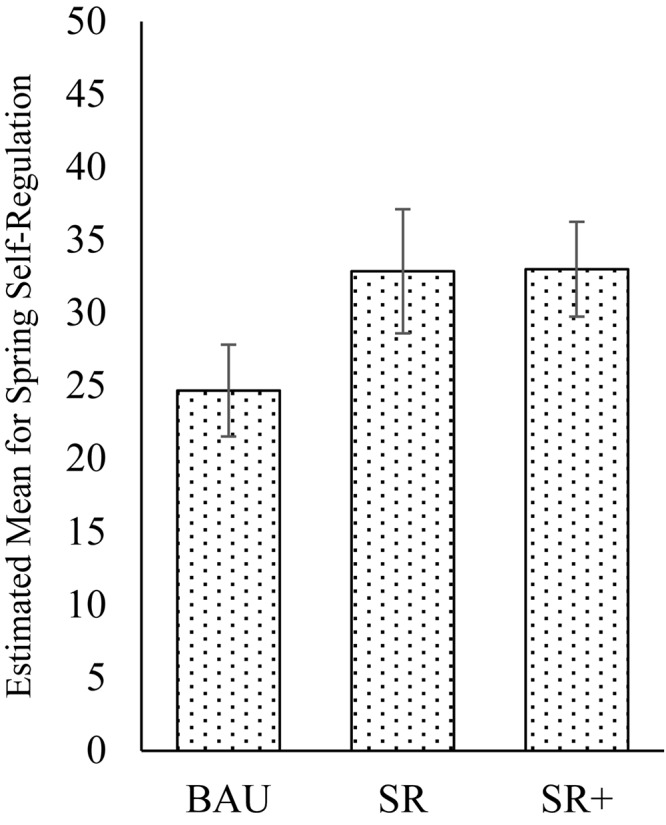
Estimated means at post-test (±SE) for self-regulation in children with low initial levels of self-regulation.

We tested an interaction between the effect of intervention type (BAU, SR, or SR+) and children with low levels of self-regulation at pre-test. Results indicated a significant interaction between the intervention groups and children’s low self-regulation status. Children with low self-regulation children in the SR group showed an additional benefit of the intervention compared to children with high self-regulation, β = 0.35, *p* = 0.037. In contrast to the analyses with the overall sample (Figure 1A), for children with low initial HTKS-R scores at pre-test, children in the SR and SR+ intervention groups showed significant gains in self-regulation over and above the BAU group at post-test. This subgroup analysis for the interaction is shown in Figure 2.

## Discussion

The goal of this study was to examine a self-regulation intervention that explicitly focused on self-regulation (attentional flexibility, working memory, and inhibitory control) and to compare the self-regulation-only version of the intervention (self-regulation; SR) with an enhanced version that included emphasis on best practices to support early math (counting and cardinality and numeral knowledge), and literacy (phonological awareness and print knowledge; SR+). We examined if there were significant effects of the self-regulation intervention (SR and SR+ versions) on children’s self-regulation and academic outcomes (early literacy and math skills) over the preschool year.

Results indicated that although not statistically significant, there was a trend for children receiving either version of the intervention to show greater improvement on a measure of self-regulation, and results for the SR+ version also demonstrated a trend toward significant improvements in self-regulation. Children receiving either version of the intervention gained significantly more in math over the preschool year compared to children in the BAU group, but there were no differences between groups on literacy performance.

### Effects of the Intervention on Self-Regulation Outcomes

The present study demonstrated that children receiving either intervention version demonstrated higher self-regulation on the HTKS-R at post-test compared to the BAU group, based on measures of effect size, but results were not statistically significant. Gains on the HTKS-R for either version and for the SR+ version over the BAU group were larger and approached significance (*d* = 0.31 and *d* = 0.32 respectively), whereas gains on the HTKS in the SR version were smaller (*d* = 0.25). Despite the small sample size, effects from either version of the intervention (*d* = 0.31) were similar to effect sizes in previous studies, which were (*d* = 0.32) in Schmitt et al. (2015) and (*d* = 0.33) in Duncan et al. (2018). The lack of significant effects may be in part due to limited power and the small sample size in the present study, but the consistency in effect sizes across studies suggests the promise of a robust intervention effect (Cumming, 2014). This research also aligns with other similar interventions documenting improvements in children’s self-regulation (Blair and Raver, 2014) and recent meta-analyses of self-regulation interventions (Pandey et al., 2018), which supports the substantive and practical significance for effects of this size (Hill et al., 2008).

### Effects of the Intervention on Early Academic Outcomes

#### Effects on Math

Children receiving either version of the self-regulation intervention had significantly higher math scores at post-test compared to children in the BAU group, which was equivalent to a 24% difference in math at the end of the preschool year. Children in the SR+ version of the intervention had significantly higher math scores at post-test compared to children in the BAU group and children in the SR version showed a similar pattern. Effect sizes for either version of the intervention (0.38) and for the two versions of the intervention were substantive (0.38 and 0.34, respectively). The size of the effects did not significantly differ by intervention version (SR+ and SR), which suggests that there is something about the cognitive complexity in the self-regulation games that promotes early math skills especially in children from low-income backgrounds (as defined by Head Start enrollment in the U.S.) rather than the addition of the math and literacy components. These results support other research on self-regulation interventions (e.g., Blair and Raver, 2014; Schmitt et al., 2015), which have found significant effects on children’s early math skills. This is also supported by research finding bidirectional relations between early math and self-regulation skills in early childhood (Schmitt et al., 2017; Cameron et al., 2019; McClelland and Cameron, 2019). The nature of the intervention games required children to pay attention to, remember, and follow increasingly complex sets of rules, which are especially important for children’s early math development (McClelland et al., 2014; Purpura et al., 2017). Overall, results from the present study suggest that self-regulation interventions can improve early math skills in children from low-income families.

#### Effects on Literacy

Differences in children’s early literacy at post-test were not statistically different between the intervention groups and the BAU group. Previous research has shown mixed effects; one study on the RLPL intervention showed an overall intervention effect on improved early literacy skills with a diverse sample of children from a range of socioeconomic backgrounds (Tominey and McClelland, 2011), but a study with a low-income sample did not find significant effects of the intervention on children’s literacy skills (Schmitt et al., 2015). It is possible that effects are present in more diverse samples of children. Another possibility is that relations between early literacy and self-regulation are weaker than relations between math and self-regulation and math in early childhood (Blair et al., 2015). In older children, however, stronger reciprocal relations have been found between complex aspects of literacy such as comprehension and self-regulation (Connor et al., 2016). The results of the present study do not clearly indicate if self-regulation interventions can improve children’s early literacy skills and more research is needed.

### Differential Intervention Effects

Previous research has pointed to the importance of examining differential intervention effects on children with low initial self-regulation and children from low-income families (McClelland et al., 2017). Results from the current study indicated that children in the intervention with low baseline levels of self-regulation measured at the fall of preschool (pre-test) made significantly greater gains in self-regulation compared to children in the BAU control group with higher self-regulation measured at pre-test. This supports other research demonstrating that children with low initial self-regulation may show stronger self-regulation gains in the RLPL intervention and other self-regulation interventions compared to children with higher baseline levels of self-regulation (Tominey and McClelland, 2011; Sasser et al., 2017). It may be that children with low initial levels of self-regulation demonstrate greater risk (e.g., have exposure to greater stress and are at risk from coming from chaotic backgrounds (Blair and Raver, 2012) and have more room to improve when participating in interventions. This idea has been called the compensatory hypothesis and suggests that targeting children with low self-regulation may be one way to support school readiness in young children from low-income families. One hypothesized explanation could be that children who showed significant gains with low scores at the beginning of the year were simply demonstrating regression to the mean. Given the use of classroom randomization, however, regression to the mean is unlikely because children in the BAU control classrooms did not show the same level of improvement over the year (Diamond and Ling, 2016). Overall, however, more research is needed to investigate and replicate these findings, especially in larger and more diverse samples of children.

### Limitations and Future Directions

Results from the present study provide additional information about the effectiveness of a self-regulation intervention on school readiness in children from low-income families, but there were limitations. First, although the study specifically focused on the iterative development of the intervention and included an RCT to evaluate the promise of the intervention, the study sample was small and had limited power given that results were clustered at the (teacher) level. Future research needs to examine effects with a larger sample. Second, the sample focused on children from families with low incomes based on research indicating that these children may especially benefit from the RLPL intervention (e.g., Schmitt et al., 2015). However, this limited our ability to generalize findings beyond children from low-income families in the U.S. and future research needs to include more diverse samples of children. Third, although random assignment was used to assign teachers (and thus classroom children) to intervention and control groups, there were baseline differences on several of the variables of interest. All models included baseline skills, but our ability to make causal inferences was limited. Although results of the present study supported previous RCT evaluations of the RLPL intervention (Tominey and McClelland, 2011; Schmitt et al., 2015; Duncan et al., 2018) more research is needed. Fourth, although we used two measures of self-regulation, we largely treated self-regulation as a unidimensional construct. Future studies need to include additional measures that explicitly tap into other aspects of EF (e.g., cognitive flexibility) to better understand generalizability of our findings to other domains of self-regulation. Expanding the number of self-regulation measures would also enable the use of latent variable approaches allowing for a more nuanced understanding of the self-regulation construct. In addition, although the literacy aspect of the intervention was focused on print knowledge and phonological awareness, the outcome measure was a more general literacy measure that broadly captured letter knowledge and decoding. Thus, there may have been more targeted intervention effects on specific aspects of literacy that were not captured by the outcome measure. Future work should consider the use of measures of each of the individual targeted components of literacy to best evaluate potential intervention effects.

Finally, the study focused on two versions of a self-regulation intervention (SR and SR+), which did not vary significantly in their impact on child outcomes. The primary difference was an enhanced emphasis on best practices to promote early math and emergent literacy skills in the SR+ version of the intervention. As Head Start centers, the early childhood programs where the RCT was conducted had significant support and emphasis on embedding best practices to support emergent literacy and early math into their daily routines. Given the existing emphasis on these skills, the difference in intervention conditions may not have been as great as it would have been in settings where there was less support to integrate these skills into daily practice.

Despite these limitations, there are a number of practical implications based on the present study. First, results of the present study largely replicated previous research on the RLPL intervention including three RCTs (Tominey and McClelland, 2011; Schmitt et al., 2015; Duncan et al., 2018). Together, results from these studies suggest that the games included in the RLPL intervention are cognitively complex and can improve children’s self-regulation and early academic outcomes. Although results were largely consistent between the SR and SR+ versions of the intervention in the present study, there was some indication that adding math and literacy components to the intervention resulted in stronger outcomes than the SR version. This possibility needs to be more rigorously tested in a larger scale study with a more diverse sample of children. These findings also point to the importance of promoting self-regulation, math, and literacy as a way to support children’s school readiness, especially in children from low-income families.

Second, results suggest that the RLPL intervention, as an example of a short-term, low-cost, and feasible intervention, can produce substantive improvements in children’s math skills, with some indication of improvements in self-regulation. The RLPL intervention required minimal training (one 3-h workshop) and materials were low-cost and readily available in most early childhood classrooms (e.g., construction paper). Moreover, the games could be embedded in teachers’ everyday curricular practice (e.g., circle times), which increased the feasibility of the intervention. These factors point to the scalability and feasibility of the intervention although more work is needed to further assess these potential benefits. Overall, the present study provides additional evidence that the RLPL intervention and similar interventions focused on self-regulation may be an effective and feasible way to improve low-income children’s school readiness skills.

## Conclusion

Results extend previous research and suggest that the RLPL intervention, which includes music and movement games, can improve children’s math scores over the preschool year. There was also evidence that the intervention resulted in gains in children’s self-regulation, especially for children with low self-regulation scores at baseline. These findings suggest that low-cost interventions, which are engaging and developmentally appropriate for young children, can improve school readiness with the potential to be scalable and practical for early childhood teachers. Interventions that focus on supporting self-regulation and school readiness can help ensure that children from low-income backgrounds enter school with the skills they need to be successful.

## Data Availability Statement

The datasets for this study will not be made publicly available because we are not allowed to share data outside the key personnel for the grant by our IRB. Requests to access the datasets should be directed to the corresponding author.

## Ethics Statement

This study was carried out in accordance with the recommendations of the Internal Review Board (IRB) at Oregon State University with written informed consent from parents and verbal assent from children. Parents gave written consent and children gave verbal assent in accordance with the Declaration of Helsinki. The protocol was approved by the IRB at Oregon State University.

## Author Contributions

MM, ST, SS, BH, DP, CG, and AT contributed to the conceptualization and design of the study. MM, ST, SS, DP, and CG contributed to the data analysis and results. All authors contributed to the writing of the manuscript.

## Conflict of Interest

The authors declare that the research was conducted in the absence of any commercial or financial relationships that could be construed as a potential conflict of interest.
